# Functional gene pyrosequencing reveals core proteobacterial denitrifiers in boreal lakes

**DOI:** 10.3389/fmicb.2015.00674

**Published:** 2015-07-01

**Authors:** Jatta Saarenheimo, Marja Annika Tiirola, Antti J. Rissanen

**Affiliations:** Department of Biological and Environmental Science, University of JyväskyläJyväskylä, Finland

**Keywords:** community composition, denitrification, *nirS*, *nirK*, *nosZ*, qPCR

## Abstract

Denitrification is an important microbial process in aquatic ecosystems that can reduce the effects of eutrophication. Here, quantification and pyrosequencing of *nirS*, *nirK*, and *nosZ* genes encoding for nitrite and nitrous oxide reductases was performed in sediment samples from four boreal lakes to determine the structure and seasonal stability of denitrifying microbial populations. Sediment quality and nitrate concentrations were linked to the quantity and diversity of denitrification genes, the abundance of denitrifying populations (*nirS* and *nosZ* genes) correlated with coupled nitrification-denitrification (Dn), and the denitrification of the overlying water NO_3_^-^ (Dw) correlated with the *nirS/nirK* ratio. The number of core *nirS*, *nirK*, and *nosZ* operational taxonomical units (OTUs) was low (6, 7, and 3, respectively), and most of these core OTUs were shared among the lakes. Dominant *nirK* sequences matched best with those of the order *Rhizobiales*, which was one of the main bacterial orders present in the sediment microbiomes, whereas the dominant *nirS* sequences were affiliated with the order *Burkholderiales*. Over half of the *nosZ* sequences belonged to a single OTU of the order *Burkholderiales*, but coupled nitrification–denitrification rate correlated with another dominant *nosZ* OTU assigned to the order *Rhodospirillales*. The study indicates that a few core proteobacterial clusters may drive denitrification in boreal lake sediments, as the same *Alpha*- and *Betaproteobacteria* denitrifier clusters were present in different lakes and seasons.

## Introduction

Denitrification is an important microbial process that removes excess nitrogen from aquatic ecosystems. This dissimilatory process reduces nitrate (NO_3_^-^) to nitrogen gas (N_2_) through intermediates of nitrite (NO_2_^-^), nitric oxide (NO) and greenhouse gas nitrous oxide (N_2_O), which is produced in the truncated denitrification pathway. Lakes are ‘hot spots’ of nitrogen removal, significantly reducing the N loading from land before it reaches vulnerable coastal ecosystems (Lepistö et al., 2006; Seitzinger et al., 2006). A meta-analysis focusing on denitrification in various aquatic environments (oceans, coastal ecosystems, estuaries, lakes and rivers) showed that nitrate concentration was the most important factor controlling the denitrification process (Piña-Ochoa and Álvarez-Conbelas, 2006). Nitrate also controls the key bacterial processes, e.g., organic matter degradation and biogeochemical cycling of various elements (Martins et al., 2011; Cheng et al., 2014), by modifying the microbial community composition (Zhang et al., 2015). The dominant phylogenetic groups in lake ecosystems are *Proteobacteria, Nitrospira, Actinobacteria, Acidobacteria*, and *Verrucomicrobia* (Newton et al., 2011; Zhang et al., 2015).

In addition to nitrate, other environmental factors have also been shown to control denitrification rates. For example, fresh carbon and oxygen cycles on a seasonal and diurnal basis were positively related to denitrification in a stream ecosystem (Tatariw et al., 2013). Besides environmental factors, denitrification activity can be linked to variations in denitrification genes. The denitrification pathway consists of four enzymatically catalyzed reductive steps that different microbes including bacteria (Zumft, 1997), archaea (Philippot, 2002) and fungi (Shoun et al., 1992; Tanimoto et al., 1992) perform. Production of the first gaseous product (NO) is catalyzed by one of two possible nitrite reductases (nir): cytochrome cd1 heme type reductase encoded by the *nirS* gene or copper-oxidoreductase encoded by *nirK* (Zumft, 1997). The *nirS* and *nirK* reductases are functionally equivalent, but structurally divergent. The enzymes are mutually exclusive in cultivated strains (Jones and Hallin, 2010). Although the abundance of these genes, like *nirS* and *nirK*, has been uncoupled to *in situ* denitrification rates in many soil studies (Dandie et al., 2008; Cuhel et al., 2010), the abundance of the genes, especially *nirS*, has been shown to correlate with potential denitrification rates (Hallin et al., 2009; Enwall et al., 2010; Graham et al., 2010). The distribution of *nirS* and *nirK*-containing denitrifiers is environment-specific (Junier et al., 2008; Jones and Hallin, 2010; Kim et al., 2011) and the relationship between *nirS* and *nirK* denitrifiers is thought to be controlled by dissolved oxygen regimes, with *nirS* favoring lower redox conditions (Knapp et al., 2009; Graham et al., 2010; Tatariw et al., 2013).

Most of the known nitrite reductase sequences are derived from bacteria of the phylum *Proteobacteria* (Jones et al., 2008), where *Alpha*-, *Beta*-, and *Gammaproteobacteria* are typical classes of denitrifiers (Vila-Costa et al., 2014; Yu et al., 2014). Linking the functional genes community composition and denitrification processes has been used to better understand the role of microbial control. Sequencing *nirS* genes from salt marsh sediments showed a high degree of endemic operational taxonomical units (OTUs) present in single samples (Bowen et al., 2013), whereas *nirS*, *nirK*, and *nosZ* communities in acidic peat soils (Palmer et al., 2012) and *nirS* and *nirK* communities in rice paddy fields (Yoshida et al., 2009) were mostly dominated by a few core OTUs. Such information from other aquatic systems, such as lakes, is lacking. However, as salinity is a very important factor shaping the structure of denitrifier communities (Jones and Hallin, 2010), one could predict that also other freshwater systems than peatlands and rice fields show similar patterns, i.e., are dominated by a few core OTUs. In addition, it is still unclear, whether the community composition or gene abundances can be used to predict the prevailing process rates in aquatic ecosystem. To our knowledge, no previous study of freshwater systems combines process measurements with analysis of both the abundance and alpha- and beta- diversity of nitrite – reducers and nitrous oxide – reducers. Thus, possible links between community structure and denitrification rates might have been previously unnoticed.

Here, we hypothesized that denitrifier community of lake ecosystems is dominated by a few core OTUs, i.e., by a low number of different bacterial groups. In addition, we hypothesized that the abundance and/or diversity of at least one of the denitrification genes (*nirS*, *nirK*, and *nosZ*) predicts denitrification rates in lakes. The study was conducted at an inter-lake scale, covering four boreal lakes in Southern Finland with varying nitrate concentrations and across a depth gradient within a single lake (Lake Ormajärvi) with a temporal (four seasons) resolution. Besides our recent DGGE study of *nirK* genes (Rissanen et al., 2013), to our knowledge, correlations of *nirS*, *nirK*, and *nosZ* genes and denitrification rates have not been analyzed from boreal lake sediments, which are important filters of anthropogenic nitrogen loads. Molecular analysis was performed by quantitative PCR (qPCR) and 454-pyrosequencing. All background environmental data and denitrification rates were previously collected (Rissanen et al., 2011, 2013), with the isotope pairing technique (IPT) that allows division of the intrinsic denitrification rates to coupled nitrification–denitrification and denitrification of the NO_3_^-^ in overlying water (Dw).

## Materials and Methods

### Sample and Data Collection

The study lakes, Ormajärvi, Pääjärvi, Suolijärvi, and Lehee, represent typical small to medium sized boreal lakes with mean depths ranging from 1 to 15 m and trophic status ranging from mesotrophic to eutrophic. Rissanen et al. (2011, 2013) gives a full description of the study lakes, sample collection, DNA extraction and measurement of environmental factors and denitrification rates (Supplementary Table S1). Briefly, for the intra-lake scale component, surface sediment samples (down to a depth of 1 cm) were collected from shallow littoral (1 m), deep littoral (3 m) and profundal sites (8 m) of Ormajärvi during four seasons (early-summer, mid-summer, autumn, and winter) in 2006–2007 (Rissanen et al., 2011). At each sampling event, three sediment cores were taken from every depth (36 samples total). For the inter-lake scale study, samples (down to a depth of 2 cm) were collected from profundal sites of Pääjärvi (10 and 12 m), Suolijärvi (10 m) and Lehee (3.3 m) during summer and autumn in 2007 (Rissanen et al., 2013) and were analyzed together with the profundal samples of Ormajärvi (Supplementary Table S1). From lakes Pääjärvi, Suolijärvi, and Lehee, three sediment cores were taken during both sampling occasions (six samples from each lake). Concurrently collected environmental data included organic content [loss on ignition (LOI)] and sediment porosity as well as temperature, [O_2_], [NO_3_^-^ + NO_2_^-^], [NH_4_^+^], and [PO_4_^3-^] from water above the sediment surface (∼10 cm for O_2_ and T; ∼2–3 cm for nutrients). Sediment denitrification rate variables (N_2_ production rate) were measured using IPT calculations (Nielsen, 1992) through incubations with ^15^NO_3_^-^ – label as the production of ^29^N_2_ and ^30^N_2_ and included Dn (natural coupled nitrification–denitrification), Dw (denitrification of the natural NO_3_^-^ in the water above the sediment) and Den (denitrification of the natural NO_3_^-^, i.e., Dn + Dw), herein referred to as total denitrification. IPT samples were measured with a mass spectrometer (Europa Scientific, Roboprep-G-Plus, and Tracermass) at the National Environmental Research Institute in Silkeborg, Denmark.

### qPCR of *nirS, nirK, and nosZ*

The abundance of *nirS*, *nirK*, and *nosZ* genes were measured with qPCR using the partial 16S rRNA gene as a reference gene. For the Lake Ormajärvi depth transect, altogether 36 samples were analyzed. For the inter-lake scale study, sample replicates from Pääjärvi, Suolijärvi, and Lehee were pooled after DNA extraction and the final set, therefore, consisted of two samples from each Pääjärvi, Suolijärvi, and Lehee, and three samples from Ormajärvi. Primer pairs used were nirSCd3aF/nirSR3cd (Kandeler et al., 2006) for *nirS*, nirK876/nirK1040 (Henry et al., 2004) for *nirK*, nosZ2F/nosZ2R (Henry et al., 2006) for *nosZ* and 27f/338r (Lane, 1991, and Universal primer) for 16S rRNA gene. Amplification of qPCR and fluorescent data collection was conducted with a Bio-Rad CFX96 thermal cycler (Bio-Rad Laboratories) following the same protocol as in Saarenheimo et al. (2015). No template inhibition was observed, when qPCR linearity was tested from original, 10- and 100-fold template dilution series. Efficiencies for PCR reactions were 87.5% for *nirS*, 92.3% for *nirK*, 89.9% for *nosZ*, and 91.6% for 16S rRNA.

### 454-Pyrosequencing

The community structure, richness, and diversity of organisms harboring 16S rRNA, *nirS*, *nirK*, and *nosZ* genes were studied using 454-pyrosequencing. This analysis was done from eight profundal samples, including early summer, mid-summer, and autumn samples from Ormajärvi, early-summer and autumn samples from Suolijärvi and Lehee and autumn sample from Pääjärvi (Supplementary Table S1). PCR was conducted with primer pairs 27F/519R (Lane, 1991 and Turner et al., 1999) for 16S rRNA, nirScd3aF/nirSR3cd (Kandeler et al., 2006) for *nirS*, F1aCu/R3Cu (Hallin and Lindgren, 1999) for *nirK* and nosZF/nosZ1622R (Kloos et al., 2001 and Throbäck et al., 2004) for *nosZ*. In the PCR reaction, 2 μl of nucleic acid extract (∼10 ng) was used as a template in a 23 μl PCR mixture containing 0.2 mM of dNTPs, 0.3 μM of each primer, 1x Biotools reaction buffer, 0.6 mg ml^-1^ BSA and 0.6 U Biotools polymerase. PCR amplification was performed in a C1000 Touch Thermal Cycler (Bio-Rad Laboratories) with an initial denaturation step at 95°C for 5 min and 35 cycles of amplification (94°C for 30 s, 53°C for 1 min, 72°C for 3 min). Barcodes that were five-base long were incorporated between the 454-Titanium adapter and the forward (*nirS*, *nirK*, and *nosZ*) or reverse (16S rRNA gene) primer to distinguish each sample in the mixed reaction. PCR products were purified with Agencourt AMPure XP purification system (Beckman Coulter, Danvers, MA, USA). PCR was done from three replicate samples (three replicate sampling cores) from each sampling event and PCR products of the replicates were pooled in equal amounts before sequencing. Finally, eight samples per each gene were sequenced (Supplementary Table S1). Sequencing was done with Titanium chemistry using a 454 GS-FLX system (454 Life Sciences, Branford, CT, USA) at the Institute of Biotechnology hosted by Helsinki University.

Barcodes and primer sequences, as well as sequences containing ambiguous nucleotides and homopolymers longer than eight nucleotides were removed from all 454 – pyrosequencing libraries using Mothur (Schloss et al., 2009). 16S rRNA gene sequences (average length = 259 bp) were de-noised with the PyroNoise – tool (Quince et al., 2009) and assigned taxonomies with the naïve Bayesian classifier – tool (based on Greengenes taxonomy, bootstrap value cut off = 80%; Wang et al., 2007) in Mothur. Sequences classified as archaea, chloroplast, mitochondria, and eukaryota were removed. Furthermore, chimeric sequences, denoted using Mothur’s implementation of Uchime (Edgar et al., 2011), were removed. Finally, 16S rRNA gene sequences were aligned in Mothur using Silva reference alignment. Protein-coding (*nirS*, *nirK*, and *nosZ*) nucleotide sequences were edited to a fixed length of 285 bp and translated into amino acid sequences, and sequences with ambiguous amino acid residues and stop codons were removed. Amino acid sequences were aligned using HMMER3 – aligner tool at FunGene (Functional gene pipeline and repository) website^1^ Sequences that were partially or completely unaligned were removed, and random samples of these unaligned sequences were compared to databases using blastp (Altschul et al., 1997) to assure that they did not represent real *nirS*/*nirK*/*nosZ* sequences. Subsequently, sequences were realigned. The respective nucleic acid sequences were checked and cleared for chimeras as explained above for 16S rRNA gene analysis. After this polishing, the final dataset contained a total of 14689, 6556, 6815, and 4735 sequences of 16S rRNA, *nirS*, *nirK*, and *nosZ* gene amplicons, respectively, corresponding to 346–2906 sequences per sample for each gene.

16S rRNA gene sequences were clustered (average neighbor method) into OTUs at the 97% similarity level and OTUs were assigned consensus taxonomies (Greengenes taxonomy, cutoff = 80%) in Mothur. For *nirS*, *nirK*, and *nosZ*, protein distance matrix (positions with gaps excluded, Kimura’s method of multiple substitutions) was calculated in ClustalX 2.0.12 (Larkin et al., 2007) and was used to cluster (average neighbor method) amino acid sequences into OTUs at the 90% similarity level in Mothur. In addition, Mothur was used to calculate number of OTUs, diversity (inverted Simpson’s diversity index), richness (Chao 1 richness estimate) and coverage (Good’s coverage), an estimate of the proportion of amplified gene amplicons represented by sequence libraries for each sample. To account for the effect of variable library sizes (variable number of sequences), these values were calculated as averages from 10000 subsamples sampled to the size of the smallest sample, which was 413, 346, and 377 sequences for *nirS*, *nirK*, and *nosZ*, respectively. To remove the possible effect of rare OTUs in multivariate analyses (see section Statistical analysis), OTUs with ≤8 sequences were removed (thus those OTUs with on average less than 1 sequence per sample were filtered out), leading to removal of 4, 6, and 3% of sequences and 58, 72, and 68% of OTUs for *nirK*, *nirS*, and *nosZ*, respectively, followed by subsampling of each sample to the size of the smallest sample. Nucleotide sequences of *nirS*, *nirK*, and *nosZ* genes were assigned taxonomies by finding the best hit from in-house databases using kmer searching in Mothur. Retrieval of sequences for these databases were done from EMBLALL-database (via SRS-server LION^2^) using search query “description = gene name (i.e., *nirS*, *nirK*, or *nosZ*) BUTNOT un,” which excluded hits from uncultured and unidentified microbes, followed by manual selection to remove duplicate sequences. Thereafter, OTUs were assigned to consensus taxonomies using Mothur (cutoff = 60%). OTUs with relative abundance >1% was used to draw a network with, where the lakes and OTUs were clustered using the spring-embedded algorithm implemented in the Cytoscape^3^ (version 3.2) (Saito et al., 2012). Seasonal changes in the core *nirS*, *nirK*, and *nosZ* OTUs in lake Ormajärvi was visualized with SigmaPlot Version 12.5. The qPCR data was used to calculate the proportion of core OTUs (*nirS, nirK*, and *nosZ*) of the whole bacterial community: qPCR abundance × OTU relative abundance. The 454-pyrosequencing data was deposited to the NCBI’s Sequence Read Archive (Accession number: Bioproject PRJNA268909 and PRJNA282004).

### Statistical Analysis

Correlations among environmental parameters, denitrification rates, functional gene abundances, combined qPCR × OTU relative abundance, inverted Simpson’s index, Chao1 richness estimate and the number of sequences in the dominant OTUs (OTUs with relative abundance >4% across all libraries) were analyzed using Spearman’s rank correlation in PASW 18.0 (PASW Statistics 18, Release Version 18.0.0, SPSS 2009). The seasonal changes in *nirS/nirK* ratio in Ormajärvi were analyzed with Kruskal–Wallis one-way analysis of variance with PASW 18.0. Multivariate analyses of the *nirS*, *nirK*, and *nosZ* community structure were based on Bray–Curtis dissimilarities calculated among samples using number of sequences in OTUs. The data was assessed graphically using non-metric multidimensional scaling (NMS) constrained to 2 ordination axes. Furthermore, UPGMA – clustering with bootstrapping (1000 replicates) was done to validate the differences among samples. The relationship between community structure and environmental factors was analyzed using a distance-based linear model (DISTLM) procedure (Anderson, 2001; McArdle and Anderson, 2001). The relationship between community structure (Bray–Curtis distances) and denitrification rates (Euclidean distances) was studied using Mantel’s test. Variables correlating significantly with the community structure were then separately correlated (Pearson) with both of the NMS axes, and the *R*^2^ values of these correlations were displayed as vectors radiating from the center of the plot (McCune and Grace, 2002). NMS and Mantel’s test were performed using PC-ORD version 6.0 (PC-ORD. Multivariate analysis of ecological data. MjM Software, Gleneden Beach, Oregon, USA; McCune and Mefford, 2011), UPGMA – clustering was done using PAST version 3.06 (Hammer et al., 2001) and DISTLM was conducted using FORTRAN program by Anderson (2004).

## Results

### Inter-Lake Scale

**FIGURE 1:**
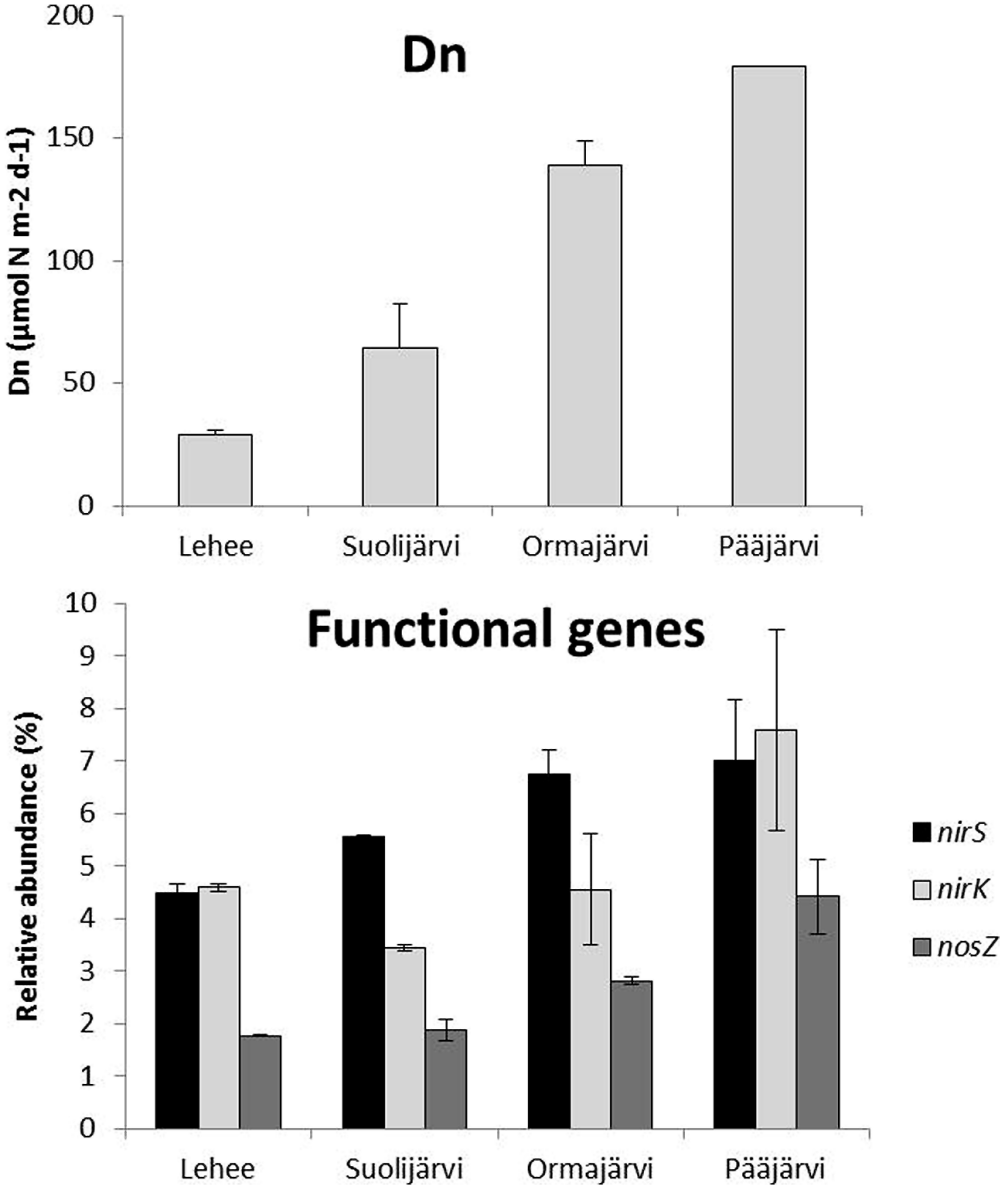
**Natural coupled nitrification–denitrification process rates (Dn) and average relative abundance of *nirS*, *nirK*, and *nosZ* genes (% of the total 16S rRNA gene abundance) in profundal sediments of four study lakes (*n* = 2 for Lehee, Suolijärvi, and, Pääjärvi and *n* = 3 for Ormajärvi)**.

Quantitative PCR analysis showed that *nirS*, *nirK*, and *nosZ* were present at all sampling sites and at every time point (varying between 4.3–8.2, 2.6–9.5, and 1.7–5.1% relative to 16S rRNA, respectively), *nirS* or *nirK* being the most abundant and *nosZ* the least abundant at all sampling sites. The highest abundances of each gene were observed in Pääjärvi during the summer season (*nirS* 8.2, *nirK* 9.5, and *nosZ* 5.1% relative to 16S rRNA; **Figure 1**), when the highest nitrate concentration was measured (76.02 μmol l^-1^, Supplementary Table S1). The *nirS* and *nosZ* gene abundances correlated positively with nitrate concentration in the water above the sediment. There was a negative correlation between porosity and the abundance of all genes, and between LOI and *nirS* and *nosZ* gene abundances (**Table 1**). The *nirS*/*nirK* ratio was on average 1.3 (±0.5). Thus, *nirS* was slightly more abundant than *nirK* in our study lakes. Although the total denitrification rates (Den) and gene abundances were uncoupled, denitrification of the overlying water NO_3_^-^ (Dw) correlated with the *nirS/nirK* ratio and coupled nitrification–denitrification (Dn) was higher with increased *nirS* and *nosZ* abundances (**Table 1**; **Figure 1**).

**Table 1:** Correlations of functional gene relative abundances (% of total 16S rRNA), inverted Simpson’s diversity index, Chao1 richness estimator with environmental parameters and measured denitrification rates (Den, total denitrification, Dn, coupled nitrification–denitrification, Dw, denitrification of the NO_3_^-^ of sediment overlying water) in the inter-lake dataset (*n* = 9).

	*nirS* %	*nirK* %	*nosZ* %	*nirS/nirK*	*nirS* Chao	*nirK* Chao	*nosZ* Chao	*nirS* InvS	*nirK* InvS	*nosZ* InvS
**Den**	–	–	–	–	–	–	–	–	–	0.9
**Dn**	**0.81**	–	**0.86**	–	–	0.81	–	0.83	–	–
**Dw**	–	–	–	0.74	–	–	–	–	–	0.9
**NO_3_^-^**	0.68	–	0.67	–	–	0.71	–	–	0.79	–
**NH_4_^+^**	–	–	–	–	–	–	–	–	–	–
**PO_4_^3-^**	–	–	–	–	–	–	–	–	0.71	–
**Temp**	–	–	–	–	–	–	–	–	–	–
**O_2_**	–	–	0.73	–	–	–	–	–	–	–
**Porosity**	**-0.85**	–0.71	**-0.9**	–	–	-0.77	–	-0.82	–	–
**LOI**	**-0.82**	–	**-0.85**	–	–	**-0.82**	–	**-0.88**	–	–

*Correlation coefficients with 0.01 < p < 0.05 are shown in plain text and coefficients where p < 0.01 are written in bold.*

Altogether, there were 388, 435, and 147 OTUs (at 90% sequence similarity level) in *nirS*, *nirK*, and *nosZ* libraries, respectively. Good’s coverage values, 0.91–0.95, 0.92–1.00, and 0.96–0.98, for *nirS*, *nirK*, and *nosZ*, respectively, indicated that the libraries were adequately large. Inverted Simpson’s diversity index values varied between 9.2–14.3, 5.3–20.0, and 2.5–4.6 for *nirS*, *nirK*, and *nosZ*, respectively. Chao richness estimates varied between 81.5–142.9, 28.0–94.5, and 27.0–64.1 for *nirS*, *nirK*, and *nosZ*, respectively. The *nirK* diversity was positively correlated with nitrate and phosphate concentrations and *nirS* diversity was positively correlated with Dn and negatively correlated with sediment LOI and porosity (**Table 1**). Correlation between total denitrification rates and gene diversities was only seen with *nosZ* (**Table 1**). *NirK* richness correlated positively with nitrate and Dn, and negatively with porosity and LOI, no other correlations among environmental factors and richness were seen (**Table 1**).

**FIGURE 2:**
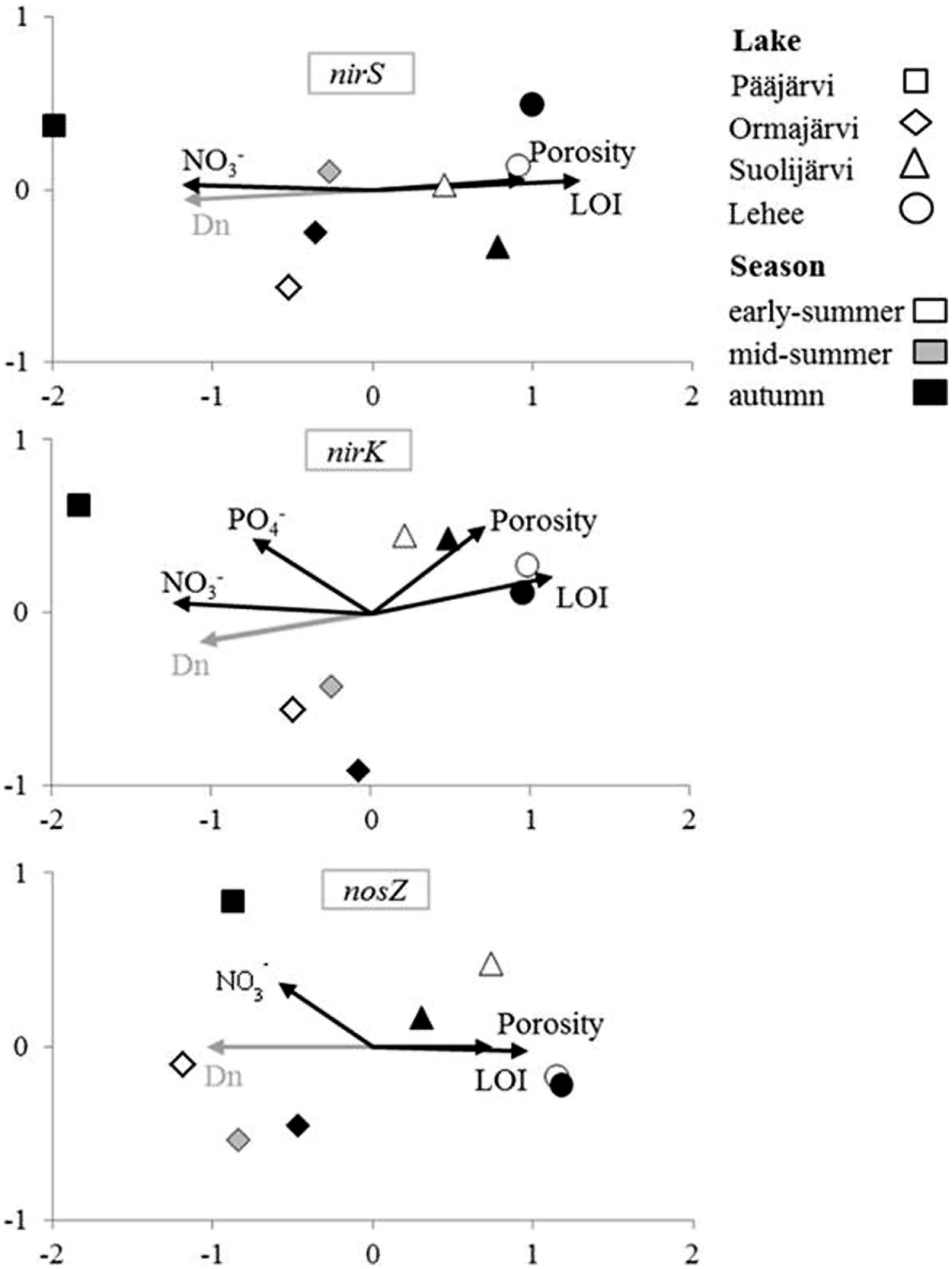
**Non-metric multidimensional scaling (NMS) analysis of environmental factors affecting denitrifier communities in the inter-lake study.** Variables correlating significantly (*p* < 0.05) with community structure in DISTLM (environmental factors) or Mantel’s test analysis (process rates) are shown by the vectors.

As visualized by the NMS analysis (**Figure 2**), *nirS*, *nirK*, and *nosZ* communities of the study lakes were distinct. The NMS axis1 explained most of the variation in all *nirS*, *nirK*, and *nosZ* communities (94.8, 84.8, and 68.6%, respectively; **Figure 2**). The highest disparity was between denitrifier communities in Pääjärvi and Lehee, which had the highest and lowest nitrate concentrations measured (**Figure 2**, Supplementary Table S1, Supplementary Figure S2). Bootstrapped cluster analysis also suggested significant differences in denitrifier communities among lakes (Supplementary Figure S2). Of the process measures only coupled nitrification–denitrification (Dn) was correlated with the community structures of each *nirS*-, *nirK*-, and *nosZ*-containing communities (Mantel’s test: *r* = 0.71 and *p* = 0.002, *r* = 0.57 and *p* = 0.001, *r* = 0.83 and *p* = 0.001, respectively). The environmental factors that correlated with community structures were nitrate concentration, porosity and LOI for all genes and phosphate for *nirK* (**Figure 2**). Environmental parameters affected the NMS community dispersion in the same direction for all genes, with nitrate and Dn affecting in the opposite direction compared to LOI and porosity (**Figure 2**). Overall the pattern for all three genes is quite similar. In addition, there was high negative inter-correlation between nitrate concentrations and LOI (*r* = -0.76 and *p* = 0.02) and positive correlation between LOI and porosity (*r* = 0.91 and *p* = 0.002).

**FIGURE 3:**
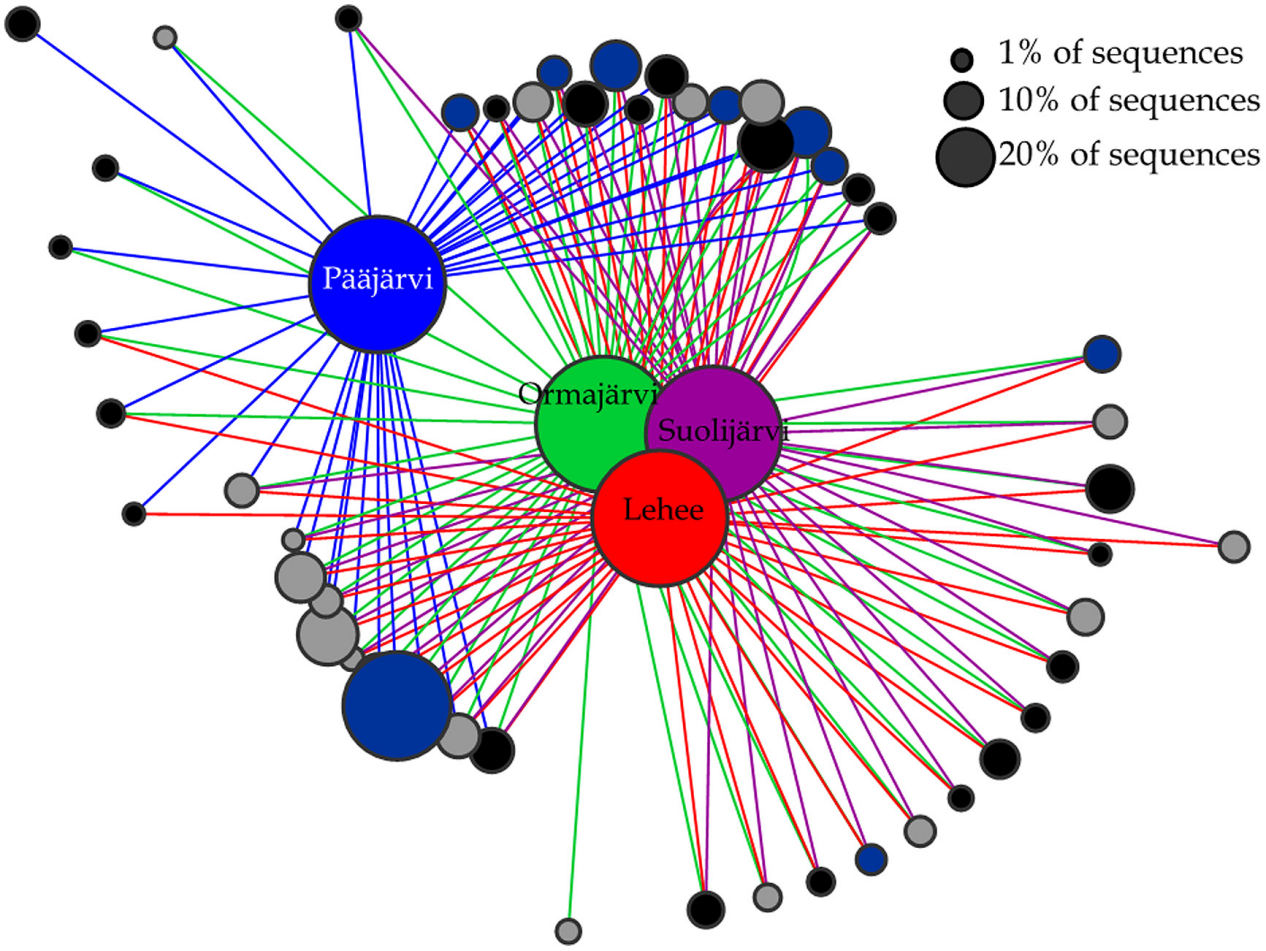
**Operational taxonomical unit (OTU)-based network analysis of *nirS* (black circles), *nirK* (gray circles), and *nosZ* (dark blue circles) sequences from lakes Pääjärvi, Ormajärvi, Suolijärvi, and Lehee.** OTUs comprising more than 1% frequency are included in the figure. Node size corresponds to the relative abundance of OTUs.

Abundant OTUs (>1% of sequences) were mostly shared among lakes (**Figure 3**). There was only one *nirK* OTU that was unique to Pääjärvi and one *nirS* OTU that was unique to Ormajärvi, while all other OTUs were found in at least two lakes (**Figure 3**). OTU based network analysis showed that lakes Ormajärvi, Suolijärvi, and Lehee had more similar communities and shared more OTUs compared to Pääjärvi (**Figure 3**), which can be partly explained by the fact that those three lakes form a connected lake chain. In addition, some OTUs were found only in Pääjärvi and Ormajärvi, where higher nitrate concentrations were found (**Figure 3**).

**FIGURE 4:**
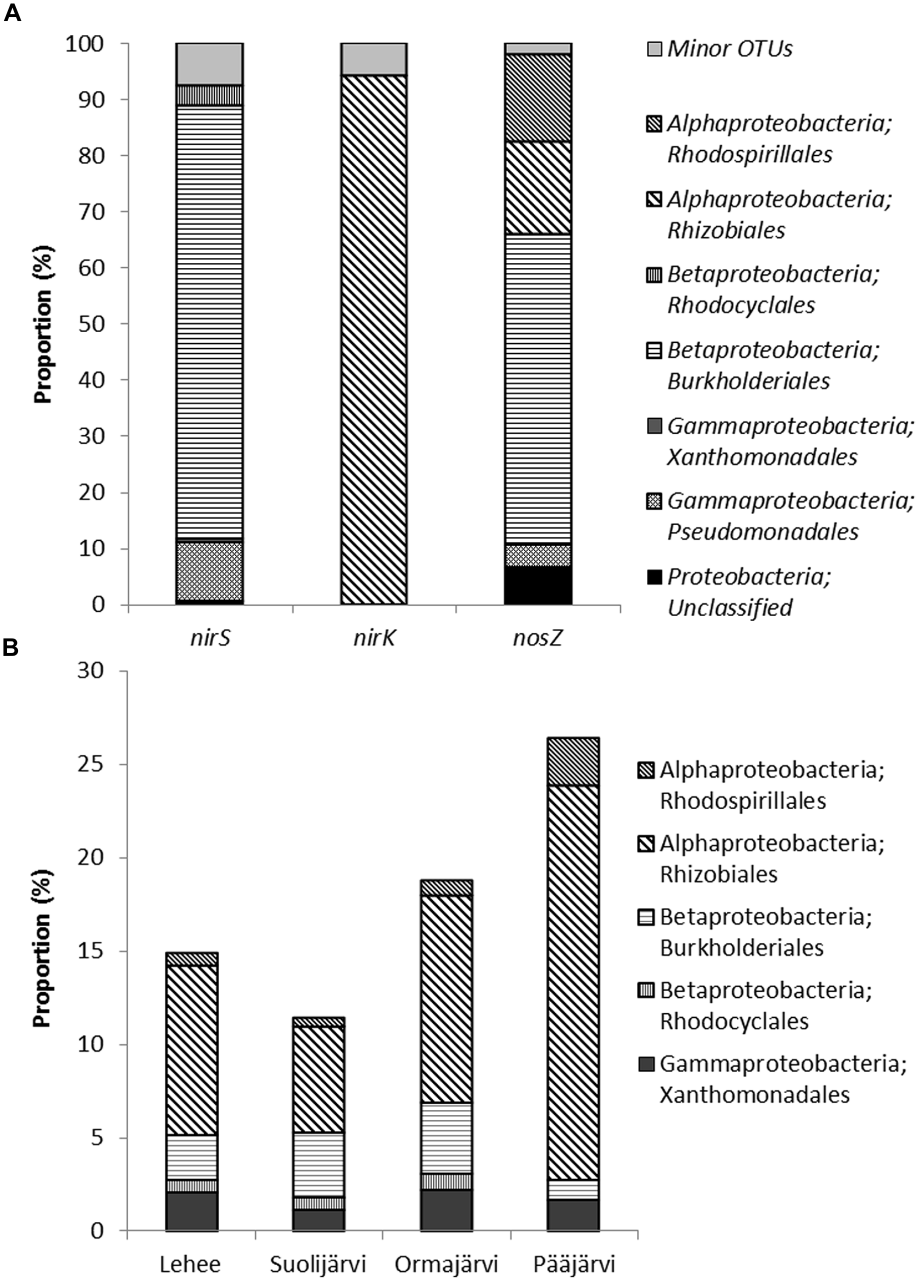
**Assignment of *nirS*, *nirK*, and *nosZ* sequences (OTUs that comprise more than 1% of sequences) to taxonomic groups **(A)**, and the proportion of these groups in the 16S rRNA data set **(B)** from the 454-pyrosequencing of 8 lake sediment samples.** Minor OTUs in **(A)** are OTUs that comprise less than 1 % of sequences.

The dominant core OTUs (with higher than 4% frequency) for *nirS*, *nirK*, and *nosZ* included only 6, 7, and 3 OTUs, while covering 57.5, 61.0, and 77.4% of all *nirS*, *nirK*, and *nosZ* sequences, respectively (Supplementary Table S2). Combined qPCR proportions and OTU relative abundances showed that the abundance of these core OTUs was 0.12–1.1% when compared to 16S rRNA gene abundance in the sediment samples (Supplementary Table S2). All 6 core *nirS* OTUs matched *Betaproteobacteria* order *Burkholderiales*, genus *Rubrivivax* sequences with 67–99% confidence level (**Figure 4**, Supplementary Table S2). Genus *Rubrivivax* was also the closest match for most of the dominant *nirS* OTUs that had 1–4% frequency. All the core *nirK* OTUs and all OTUs with >1% frequency matched sequences of *Alphaproteobacteria* order *Rhizobiales*, genus *Ochrobactrum* with a confirmed 100% confidence level (**Figure 4**, Supplementary Table S2). The three core *nosZ* OTUs belonged to *Betaproteobacteria* order *Burkholderiales* (genus *Achromobacter* but only 67% confidence level, 53.0% of sequences) and to *Alphaproteobacteria* order *Rhizobiales* (genus *Ochrobactrum*, 12.8%) and *Rhodospirillales* (genus *Azospirillum*, 11.6%; **Figure 4**, Supplementary Table S2). The core OTUs of *nirS, nirK*, and *nosZ* showed different correlations patterns where nitrate and sediment properties LOI and porosity were found to be important (Supplementary Table S2). From the process rate measurements only coupled nitrification–denitrification showed strong positive correlation with one *nirS* OTU assigned to *Burkholderiales* and *nosZ* OTU belonging to *Rhodospirillales* (Supplementary Table S2). The combined qPCR abundance × OTU relative abundance data showed somewhat similar correlations as the relative core OTU data (Supplementary Table S3). Overall, it is interesting that phosphate correlated only with *nirK* OTUs, both when observing core OTUs and the combined qPCR × OTU data, and not with any *nirS* or *nosZ* OTUs (Supplementary Tables S2 and S3).

The sediment 16S rRNA based phylogenetic classification showed that the sediment microbial communities were dominated by *Proteobacteria* (33–39% of all sequences). The other main phylogenetic groups were *Chloroflexi* (21–24% of all sequences), *Actinobacteria* (9–14%), *Nitrospirae* (8-12 %), *Acidobacteria* (4–6%), *Verrucomicrobia* (3–4%), *Gemmatimonadetes* (1–2%), and *Bacteroidetes* (1–3%), (Supplementary Figure S1). The same orders of *Alpha*- and *Betaproteobacteria* were found as was based on the functional gene classification (**Figure 4**, Supplementary Figure S1) in all four study lakes. On average the order *Rhizobiales* covered 11% of the 16S rRNA community (**Figure 4**) and the calculated proportion of denitrifying *Rhizobiales* was 2.9% (calculated from the combined qPCR*OTU abundance). The order *Burkholderiales* was only covering on average 2.7% of the 16S rRNA community, whereas the calculated proportion of denitrifiers was higher 3.8%.

### Lake Ormajärvi Depth Transect

Average relative abundances of *nirS*, *nirK*, and *nosZ* were 7.9% (±2.2%), 5.7% (±2.8%), and 2.8% (±0.9%), respectively, following a similar pattern as found at the inter-lake scale, with *nirS* slightly more abundant than *nirK*, and *nosZ* being least abundant in all samples (Supplementary Figure S3). *NirS*, *nirK*, and *nosZ* abundances correlated positively with nitrate (*r* = 0.44 and *p* = 0.007, *r* = 0.36 and *p* = 0.034, and *r* = 0.66 and *p* < 0.001, respectively). In addition, *nirS* and *nosZ* had a negative correlation with temperature (*r* = -0.44 and *p* = 0.007 and *r* = -0.49 and *p* = 0.002, respectively) and *nosZ* gene abundances further correlated positively with LOI and porosity (*r* = 0.51 and *p* = 0.002 and *r* = 0.48 and *p* = 0.003, respectively). At the intra-lake scale, we could not see any correlation between measured process rates and gene abundances (*p* > 0.05).

**FIGURE 5:**
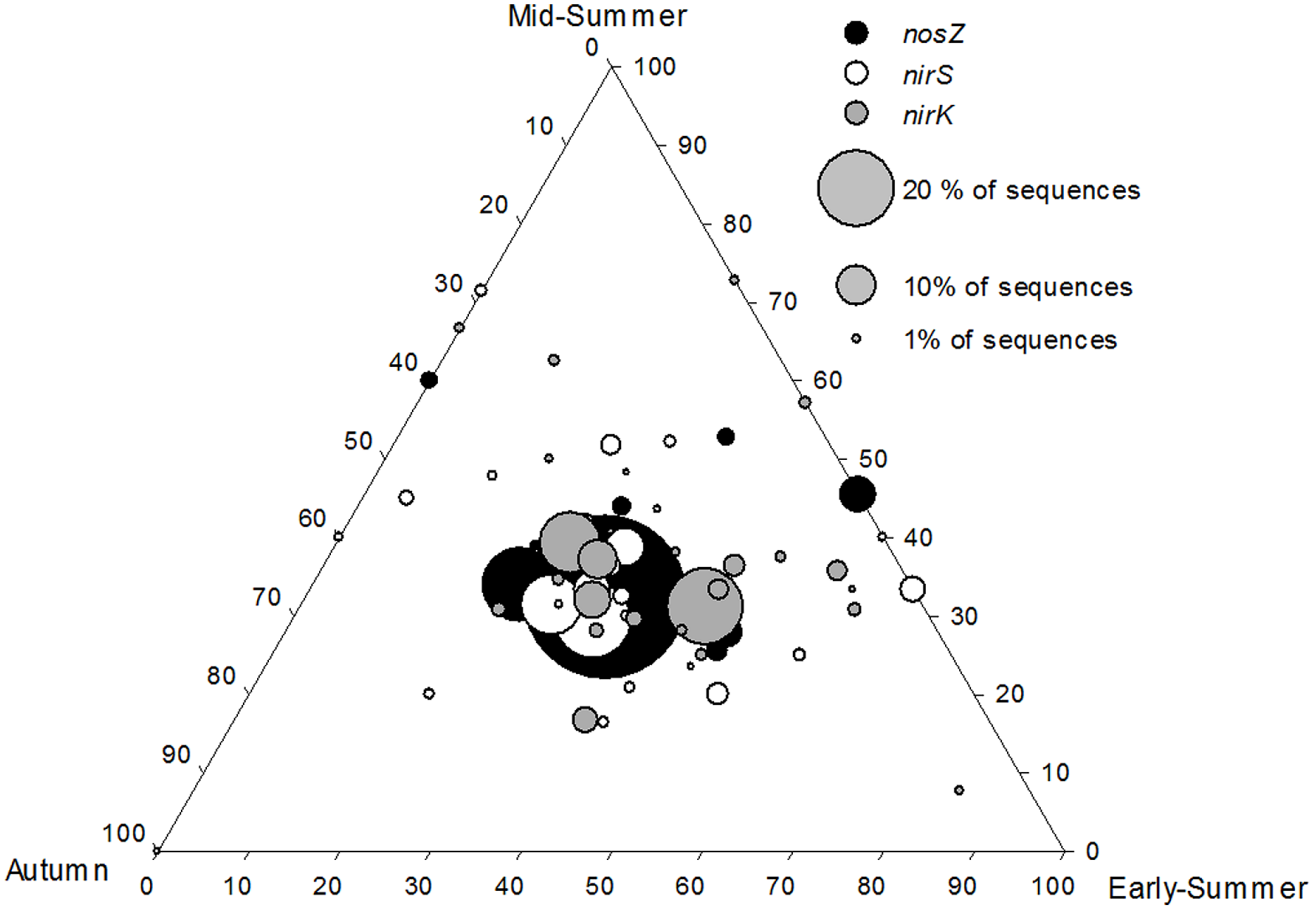
**Ternary plot showing that core *nirS*, *nirK*, and *nosZ* OTUs were equally abundant during the three seasons (early-summer, mid-summer, and autumn) at the profundal site (8 m) of Lake Ormajärvi, placing OTU symbols in the middle of the plot.** OTUs comprising more than 1% frequency are included. Node size corresponds to the relative abundance of OTUs.

Along the depth transect in Lake Ormajärvi, the *nirS*/*nirK* ratio decreased as autumn and winter approached (Supplementary Figure S4). This stemmed from the increase in relative abundance of *nirK*, whereas the proportion of *nirS* remained stable. Environmental parameters were not linked to the changes in the *nirS/nirK* ratio (Spearman correlations, *p* > 0.05), but season was shown to be an important factor affecting this ratio (Kruskal–Wallis *H* = 16.16 and *p* = 0.001). In the pairwise comparisons, the early summer *nirS*/*nirK* ratio differed significantly from autumn and winter ratios (*p* = 0.004 and *p* = 0.01, respectively). When studying the seasonal effect at different depths, season correlated with the *nirS*/*nirK* ratio at the Littoral 1 m site and at the Profundal 8 m site (*H* = 9.97 and *p* = 0.019, *H* = 9.67 and *p* = 0.027, respectively), whereas the deep Littoral (3 m) site did not show the same pattern in the *nirS/nirK* ratio (Supplementary Figure S4). The community composition did not differ between seasons at the Profundal 8 m site (**Figure 5**), but the OTUs (more abundant than 0.5% of all sequences) of *nirS*, *nirK*, and *nosZ* were observed at all times (early-summer, mid-summer, and autumn). However, none of the OTUs were present only during early-summer and autumn, whereas some OTUs were only detected in early- and mid-summer, or at mid-summer and autumn (**Figure 5**).

## Discussion

Freshwater sediments account for about 20% of total global denitrification (Seitzinger et al., 2006), with this process mainly taking place below the oxic–anoxic interface. Our earlier study with the IPT technique showed a tight connection between hypolimnetic nitrate concentration and total denitrification (Den) in sediment, as well as a relationship between nitrate and the two components of denitrification (Dn and Dw) in boreal lake ecosystems (Rissanen et al., 2013). The results of this study showed that nitrate concentration and coupled nitrification–denitrification were also connected to the relative abundance (*nirS* and *nosZ*), beta-diversity (*nirS, nirK*, and *nosZ*) and richness (*nirK*) of denitrifiers. However, against our hypothesis, the total denitrification rate could not be predicted by the abundance or community structure of denitrifiers.

The two functionally equivalent nitrite reductases (*nirS* and *nirK*) have different responses to environmental factors (Junier et al., 2008; Jones and Hallin, 2010), which was also seen in some small differences in our dataset. Phosphate concentrations were negatively correlated with *nirK* OTUs, whereas the nitrate concentration could only explain the *nirS* gene abundance. Earlier reported oxygen control on *nirS* and *nirK*, where *nirS* is thought to favor lower redox conditions (Tatariw et al., 2013) was not observed here. In our lake sediments, *nirS* was slightly more abundant than *nirK* (the *nirS/nirK* ratio was 1.3) and the relative abundance of these genes was at the same level as reported in previous studies (Ducey et al., 2011; Saarenheimo et al., 2015). However, the relative abundance of denitrifying genes may be higher, as prokaryotes can have more than one 16S rRNA operons, as many as 15 copies (Klappenbach et al., 2001). These numbers are comparable to the relative abundances reported, for example, in an anaerobic wastewater lagoon (1.3% for *nirS*, 5.3% for *nirK* and 0.3% for *nosZ* gene) by Ducey et al. (2011). Here and elsewhere, reported *nosZ* genes have been lower than the sum of *nirS* and *nirK*, but it has to be remembered that traditional *nosZ* primers may cover only part of the *nosZ* populations. Recent studies have revealed a new *nosZ* clade, which is not amplified with traditional primers (Sanford et al., 2012; Jones et al., 2013). We recently elucidated the role of this new *nosZ* clade in lake sediments (Saarenheimo et al., 2015) and it is not in the scope of this study.

Sediment properties (LOI and porosity) were negatively correlated with the relative abundance of functional genes (especially *nirS* and *nosZ*) and affected the structure of the microbial communities, which is consistent with results from the Jiazhou Bay (China) sediment study, in which silt content shaped *nirS* communities (Dang et al., 2009). The negative correlation between LOI of sediment and NO_3_^-^ concentration of the overlying water suggests that in lakes with lower LOI, higher mineral matter content may promote nitrification, which releases NO_3_^-^ to the overlying water and promotes denitrification as well as certain *nirS* and *nosZ* communities. The abundance of denitrifying populations (*nirS* and *nosZ* genes) also correlated with coupled nitrification–denitrification (Dn), which is actually the dominating component of denitrification in boreal lakes, where nitrate concentrations in the overlying water are relatively low (Rissanen et al., 2013). A tight correlation between nitrification and denitrification has been suggested by Seitzinger et al. (2006), and was further supported by the coupling between *nir* genes and *amoA* genes (responsible for nitrification) in epilithic biofilms (Vila-Costa et al., 2014). In addition the relatively high proportion of genus *Nitrospira* in the sediment sequences, which is connected to nitrite–oxidation step in nitrification (Bock and Koops, 1992), further supports the findings and indicates the importance for further studies on understanding the role of nitrification in boreal lake sediment.

As hypothesized, denitrifier community consisted dominantly by a low number of core *nir* and *nos* OTUs. This suggests that denitrification was actually driven by a selected group of bacteria, which was shared between the study lakes, even though nitrate and oxygen concentrations varied. The same core denitrifier groups were found at different lakes and seasons indicating that the process can be driven by only a few proteobacterial groups. However, the total *nirS*, *nirK*, and *nosZ* communities were distinct in each lake, suggesting that the less-dominant OTUs were spatially unique, as has previously been shown in aquatic ecosystems (Junier et al., 2008; Kim et al., 2011). This may indicate that less abundant OTUs were more affected by nitrate concentrations and sediment properties, which modified community patterns in each lake. Geographic location and water quality played a role, as physically connected lake systems had more similar communities. A fertilization study in a salt marsh also showed that higher nutrient concentrations can increase the number of unique denitrifier OTUs (Bowen et al., 2013).

The results of the main phylogenetic groups found from the boreal lake sediments are in concurrence with previous findings from lake sediments (13 lakes in China; Zhang et al., 2015) and from lake water column (69 published studies across different continents; Newton et al., 2011), where in addition to *Nitrospira* other main groups were *Proteobacteria, Chloroflexi*, and *Actinobacteria*. The most abundant *nirS*, *nirK*, and *nosZ* sequences all matched sequences of the phylum *Proteobacteria*. These taxonomic divisions followed the same patterns that have been previously reported for denitrifiers (Jones et al., 2008; Vila-Costa et al., 2014), where the most abundant *nirS* sequences matched *Betaproteobacteria* and all of the most abundant *nirK* sequences matched *Alphaproteobacteria*. Interestingly, Yoshida et al. (2009) reported that the same microbial orders (*nirS* – *Burkholderiales* and *Rhodocyclales*, *nirK* – *Rhizobiales*) found in our study lakes dominate rice paddy field soils. This may either be a general phenomenon or due to the use of the exact same primer pair in the PCR analysis. All core *nirS* OTUs were closest to sequences of the genus *Rubrivivax*. *Rubrivivax gelatinosum* is a facultative photoheterotrophic bacterium harboring both *nirS* and *nosZ* genes, but lacking nitrate reductase. Therefore, it can reduce NO_2_^-^ to N_2_ gas, but cannot use NO_3_^-^ as a substrate for the denitrification pathway (Nagashima et al., 2012). Thus, it depends on NO_2_^-^ supplied by denitrifiers and other NO_3_^-^ – reducers, which might underlie the positive correlation between NO_3_^-^ and *nirS* abundance: with higher availability of NO_3_^-^, a relatively higher proportion of NO_2_^-^ produced in NO_3_^-^ – reduction could be available. However, NO_2_^-^ is also produced by nitrifiers (NH_4_^+^ – oxidizers), which might underlie the positive correlation between Dn and *nirS* abundance. All relevant *nirK* sequences (with >1% frequency) were taxonomically closest to the genus *Ochrobactrum*, which contains species with both the nitrogen-fixation and denitrification pathways (Lee and Park, 2009). The co-occurrence of denitrification and nitrogen fixation has been previously reported in aquatic systems (Halm et al., 2009; Fernandez et al., 2011).

Many microbes harboring *nir* genes (especially *nirK* genes) have truncated denitrification pathways and are, thus, lacking the *nosZ* gene that is responsible for the last reduction step in denitrification (Jones et al., 2008). Here, the single most abundant *nosZ* OTU covered 53% of sequences, which would suggest a unique group of nitrous oxide reducers in boreal lakes. This OTU was taxonomically affiliated with *Burkholderiales*, but to a different group than the dominant *nirS* sequences. However, organisms carrying dominant *nirS* and *nosZ* genes may possibly be the same, despite being split taxonomically, as uncertainties in taxonomic classification and possible horizontal gene transfer complicates the analysis of functional genes (Jones et al., 2008). Furthermore, all the PCR based methods may suffer from primer biases, which we cannot rule out in this study. It was very recently shown that both commonly used *nirS* and *nirK* primer pairs selected for this study would underestimate the phenotypic diversity of denitrifiers, especially Gram-positive denitrifiers (Verbaendert et al., 2014), whereas proteobacterial species are favored (Penton et al., 2013; Verbaendert et al., 2014). However, the observed high abundance of denitrifying genes in qPCR and the dominance of *Proteobacteria* in the 16S rRNA sequence libraries suggest that a substantial proportion of the potential denitrifiers were targeted with the primers selected here. At least, these primers would target the most relevant proteobacterial denitrifiers (Jones et al., 2008; Green et al., 2010), and thus provide a solid foundation for future studies.

Denitrification rates have been shown to vary between different seasons (e.g., Ahlgren et al., 1994; Rissanen et al., 2011) and higher temperatures are known to increase the process rates (Saunders and Kalff, 2001). In our study at the intra-lake scale, gene abundances varied between different seasons. The *nirS/nirK* ratio was significantly higher during early-summer compared to autumn and winter due to increasing *nirK* abundance. The opposite temporal abundance patterns was reported in a soil study by Dandie et al. (2008), where the abundance of *nirK*-carrying denitrifiers declined during the study period from early-summer to autumn. However, in both studies, the actual denitrification rates were mostly controlled by nitrogen availability, and were uncoupled from the denitrifier community abundance. The ternary plot of *nirS*, *nirK*, and *nosZ* communities (**Figure 5**) shows the seasonal stability in the relative frequencies of core OTUs, indicated by the largest symbols positioned in the middle of the plot. This is in accordance with results of replicated *nirK* – DGGE data (*n* = 3 per each season) of the same samples (Rissanen et al., 2011). Thus, the results show that the core community composition remains stable even if the total abundance changes during the season

## Conclusion

This study suggests that total denitrification was dependent on short-term environmental factors, as denitrifying genes did not explain the total process rates. However, both the abundance and diversity of denitrifying genes was linked to the mineral content of the sediment, as well as to nitrification–denitrification and further to nitrate concentration in the overlying water. Although potential primer biases and uncertainties in the taxonomic assignment of functional genes complicates interpretation, the dominant *nirS*, *nirK*, and *nosZ* sequences were all assigned to *Proteobacteria*, consisting of only a few core OTUs that were widely present in the study lakes. Core *nirS* sequences were affiliated to *Rubrivivax*, which lacks nitrate reductase and needs external nitrite for denitrification. Whether the lack of upstream genes in some *nir* populations explains seasonal and spatial distributions of *nirS* and *nirK* genes in lake sediments needs to be further studied.

## Conflict of Interest Statement

The authors declare that the research was conducted in the absence of any commercial or financial relationships that could be construed as a potential conflict of interest.

## Footnotes

1 http://fungene.cme.msu.edu/FunGenePipeline

2 http://www.dkfz.de/srs

3 http://www.cytoscape.org/
