# Supplementary material for: Functional gene pyrosequencing reveals core proteobacterial denitrifiers in boreal lakes
Source: Front Microbiol. 2015 Jul 1;6:674. doi: 10.3389/fmicb.2015.00674 (PMC4486872; doi:10.3389/fmicb.2015.00674)
Supplement: Supplementary file 1 [file Data_Sheet_1.DOCX]

***Supplementary material***

**Functional gene pyrosequencing reveals core proteobacterial denitrifiers in boreal lakes**

**Jatta Saarenheimo^1^*, Marja Tiirola^1^, Antti J. Rissanen^1^**

^1^ University of Jyväskylä, Department of Biological and Environmental Science, P.O.Box 35, 40014 Jyväskylä, Finland

*** Correspondence:** Jatta Saarenheimo, Department of Biological and Environmental Science, University of Jyväskylä, P.O.Box 35, 40014 Jyväskylä, Finland.

[jatta.saarenheimo@jyu.fi](mailto:jatta.saarenheimo@jyu.fi)

**Supplementary Table 1.** Study site information and description of samples included in the molecular analysis. Hypolimnetic nutrient concentrations, oxygen status and sediment characteristics of the studied lakes. Sediment denitrification rate variables (N_2_ production rate), including Dn (natural coupled nitrification-denitrification), Dw (denitrification of the natural NO_3_^-^ in the water above the sediment) and Den (denitrification of the natural NO_3_^-^, combines Dn and Dw results). All background environmental data and denitrification rates were previously collected and reported in Rissanen et al. (1, 2).

| **Site** | **Season** | **Depth** | **qPCR** | **454-sequencing** | **Denitrification (IPT)** | **Den**  **(µmol N m^-2^ d^-1^)** | **Dn**  **(µmol N m^-2^ d^-1^)** | **Dw**  **(µmol N m^-2^ d^-1^)** | **[NO_3_^-^]**  **(µmol l^-1^)** | **[NH_4_^+^]**  **(µmol l^-1^)** | **[PO_4_^3-^]**  **(µmol l^-1^)** | **T**  **(°C)** | **O_2_**  **(µmol l^-1^)** | **Porosity** | **LOI**  **(%)** |
| --- | --- | --- | --- | --- | --- | --- | --- | --- | --- | --- | --- | --- | --- | --- | --- |
| Ormajärvi 2006 | Early-summer | LIT 1m | x |  | x | 234.61  (±37.7) | 49.04  (±23.0) | 185.57  (±30.7) | 23.64 | 1.21 | 0.10 | 10.59 | 362.50 | 0.88 | 5.32 |
| Ormajärvi 2006 | Early-summer | LIT 3m | x |  | x | 124.02  (±35.7) | 69.71  (±34.2) | 54.31  (±8.1) | 18.21 | 1.21 | 0.23 | 14.46 | 359.38 | 0.93 | 11.22 |
| Ormajärvi 2006 | Early-summer | PROF 8m | x | x | x | 219.32  (±67.4) | 125.04  (±25.7) | 94.28  (±41.8) | 28.07 | 2.79 | 0.13 | 11.16 | 281.30 | 0.94 | 11.77 |
| Ormajärvi 2006 | Mid-summer | LIT 1m | x |  | x | 160.83  (±18.2) | 86.81  (±7.5) | 74.03  (±12.7) | 3.07 | 1.21 | 0.03 | 21.25 | 300.00 | 0.85 | 5.45 |
| Ormajärvi 2006 | Mid-summer | LIT 3m | x |  | x | 201.40  (±3.7) | 152.36  (±13.8) | 49.04  (±11.8) | 3.14 | 1.07 | 0.03 | 20.77 | 287.50 | 0.94 | 9.22 |
| Ormajärvi 2006 | Mid-summer | PROF 8m | x | x | x | 560.79  (±22.3) | 157.27  (±20.0) | 403.52  (±30.2) | 35.07 | 7.64 | 0.03 | 10.92 | 193.75 | 0.94 | 11.56 |
| Ormajärvi 2006 | Autumn | LIT 1m | x |  | x | 112.67  (±89.7) | 59.71  (±63.2) | 52.95  (±30.2) | 3.09 | 1.60 | 0.03 | 12.63 | 318.75 | 0.87 | 6.11 |
| Ormajärvi 2006 | Autumn | LIT 3m | x |  | x | 125.78  (±9.9) | 35.82  (±13.6) | 89.96  (±4.2) | 8.07 | 4.71 | 0.10 | 13.01 | 275.00 | 0.92 | 9.54 |
| Ormajärvi 2006 | Autumn | PROF 8m | x | x | x | 201.33  (±84.1) | 134.72  (±88.1) | 66.61  (±4.1) | 7.48 | 2.90 | 0.06 | 12.19 | 320.87 | 0.92 | 9.82 |
| Ormajärvi 2006 | Winter | LIT 1m | x |  | x | 299.73  (±31.0) | 216.53  (±44.8) | 83.19  (±17.1) | 34.29 | 0.71 | 0.16 | 0.53 | 453.10 | 0.86 | 6.52 |
| Ormajärvi 2006 | Winter | LIT 3m | x |  | x | 216.19  (±46.2) | 154.04  (±86.4) | 62.15  (±48.3) | 36.07 | 0.93 | 0.16 | 1.35 | 412.50 | 0.93 | 9.27 |
| Ormajärvi 2006 | Winter | PROF 8m | x |  | x | 185.54  (±40.9) | 108.43  (±75.3) | 77.11  (±35.4) | 36.14 | 0.93 | 0.29 | 1.47 | 378.10 | 0.94 | 11.46 |
| Suolijärvi 2007 | Summer | 10m | x | x | x | 215.79 | 46.45 | 169.34 | 17.76 | 16.33 | 0.13 | 11.4 | 62.5 | 0.96 | 16.08 |
| Suolijärvi 2007 | Autumn | 10m | x | x | x | 216.10 | 82.62 | 133.48 | 13.79 | 5.71 | 0.12 | 9.9 | 309 | 0.96 | 16.21 |
| Lehee  2007 | Summer | 3.3m | x | x | x | 44.84 | 30.81 | 14.03 | 1.79 | 0.69 | 0.05 | 17.6 | 263 | 0.96 | 18.68 |
| Lehee  2007 | Autumn | 3.3m | x | x | x | 62.76 | 27.36 | 35.40 | 2.62 | 2.31 | 0.10 | 11.8 | 319 | 0.96 | 18.20 |
| Pääjärvi 2007 | Summer | 12m | x |  | x |  |  |  | 76.02 | 1.57 | 0.14 | 10.9 | 340 | 0.93 | 8.97 |
| Pääjärvi 2007 | Autumn | 10m | x | x | x | 260.45 | 179.31 | 81.15 | 64.67 | 1.60 | 0.29 | 11.7 | 341 | 0.93 | 8.08 |

References:

1. **Rissanen AJ, Tiirola M, Ojala A**. 2011. Spatial and temporal variation in denitrification and in the denitrifier community in a boreal lake. Aquat Microb Ecol **64**:27–40.

2. **Rissanen AJ, Tiirola M, Hietanen S, Ojala A.** 2013. Interlake variation and environmental controls of denitrification across different geographical scales. Aquat Microb Ecol **69**:1–16.

**Supplementary Table 2.** Correlations of core *nirK*, *nirS* and *nosZ* OTUs (abundancy > 4% in corresponding pyrosequencing library), environmental parameters and measured denitrification rates (Den = total denitrification, Dn = coupled nitrification-denitrification, Dw = denitrification of the NO_3_ of sediment overlying water) in the inter-lake dataset (n=8). *Proteobacteria* class where each OTU was affiliated is indicated: α = *Alphaproteobacteria* and β = *Betaproteobacteria*. Relative abundance of OTU sequences in the pooled dataset of each gene is indicated as %. Confidence of functional gene phylogenetic classification is indicated as %. The core OTUs abundance from the whole bacterial community based on combined qPCR results and OTU abundance. Correlation coefficients with 0.01 < p < 0.05 are shown in plain text and coefficients where p < 0.01 are written in bold.


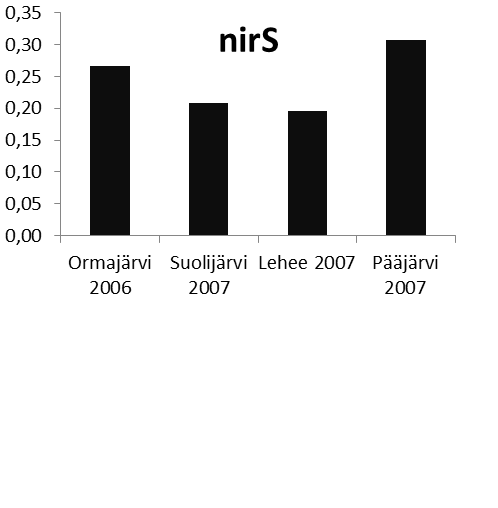

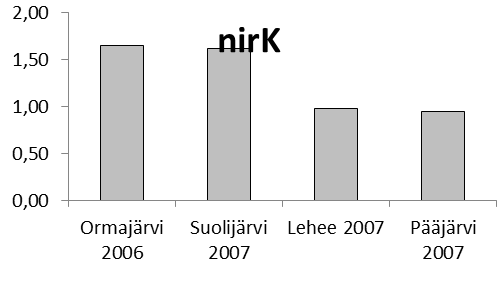

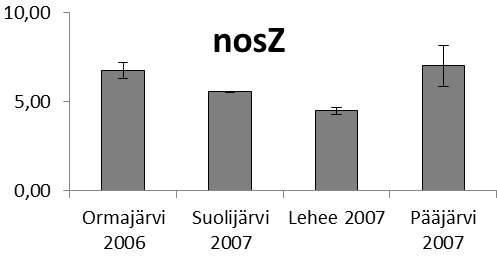


|  | ***nirK***  **OTU**  **293** | ***nirK***  **OTU**  **355** | ***nirK*OTU240** | ***nirK*OTU405** | ***nirK***  **OTU**  **259** | ***nirK***  **OTU**  **002** | ***nirK*OTU028** | ***nirS***  **OTU**  **085** | ***nirS***  **OTU**  **166** | ***nirS***  **OTU**  **359** | ***nirS***  **OTU**  **010** | ***nirS***  **OTU**  **187** | ***nirS***  **OTU**  **253** | ***nosZ*OTU**  **138** | ***nosZ***  **OTU**  **104** | ***nosZ***  **OTU**  **040** |
| --- | --- | --- | --- | --- | --- | --- | --- | --- | --- | --- | --- | --- | --- | --- | --- | --- |
| **Proteobacteria class*** | α^1^ | α^1^ | α^1^ | α^1^ | α^1^ | α^1^ | α^1^ | β^2^ | β^2^ | β^2^ | β^2^ | β^2^ | β^2^ | β^2^ | α^1^ | α^3^ |
| **Confidence (%)** | 100 | 100 | 100 | 100 | 100 | 100 | 100 | 97 | 100 | 82 | 86 | 91 | 96 | 67 | 90 | 86 |
| **Relative abundance (%)** | 18.0 | 11.5 | 9.0 | 6.6 | 6.4 | 5.4 | 4.2 | 21.0 | 9.8 | 9.2 | 6.5 | 6.4 | 4.6 | 53.0 | 12.8 | 11.6 |
| **Combined qPCR and OTU abundance (%)** | 0.62 | 0.40 | 0.31 | 0.23 | 0.22 | 0.19 | 0.14 | 1.03 | 0.48 | 0.46 | 0.32 | 0.32 | 0.23 | 1.11 | 0.27 | 0.24 |
| **Den** | - | - | - | -0.71 | - | - | - | - | -0.83 | - | - | - | - | - | -0.83 | - |
| **Dn** | - | - | -0.76 | - | -0.83 | - | - | - | -0.83 | 0.76 | - | - | - | - | **-0.93** | **0.92** |
| **Dw** | - | - | - | - | - | - | - | - | - | - | - | - | - | - | - | - |
| **NO_3_^-^** | - | - | - | -0.83 | - | - | - | - | **-0.93** | - | - | - | - | - | **-0.86** | - |
| **NH_4_^+^** | - | - | - | - | - | - | - | - | - | - | - | - | - | - | - | - |
| **PO_4_^3-^** | - | -0.74 | - | -0.78 | - | - | - | - | - | - | - | - | - | - | - | - |
| **°C** | - | - | - | - | - | - | - | - | - | - | - | - | - | - | - | - |
| **O_2_** | - | - | - | - | - | - | - | - | - | - | - | - | - | - | - | - |
| **Porosity** | - | - | **0.91** | - | 0.80 | - | - | - | - | -0.82 | - | - | 0.83 | 0.73 | 0.78 | **-0.91** |
| **LOI** | - | 0.81 | **0.88** | - | 0.77 | - | - | - | 0.81 | -0.79 | - | - | - | - | **0.86** | **-0.90** |

* Affiliated to order 1*Rhizobiales*, 2*Burkholderiales* and 3*Rhodospirillales.*

**Supplementary Table 3.** Correlations of combined qPCR abundance x core OTU relative abundance, environmental parameters and measured denitrification rates (Den = total denitrification, Dn = coupled nitrification-denitrification, Dw = denitrification of the NO_3_ of sediment overlying water) in the inter-lake dataset (n=8). Correlation coefficients with 0.01 < p < 0.05 are shown in plain text and coefficients where p < 0.01 are written in bold.

|  | ***nirK***  **OTU**  **293** | ***nirK***  **OTU**  **355** | ***nirK*OTU240** | ***nirK*OTU405** | ***nirK***  **OTU**  **259** | ***nirK***  **OTU**  **002** | ***nirK*OTU028** | ***nirS***  **OTU**  **085** | ***nirS***  **OTU**  **166** | ***nirS***  **OTU**  **359** | ***nirS***  **OTU**  **010** | ***nirS***  **OTU**  **187** | ***nirS***  **OTU**  **253** | ***nosZ*OTU**  **138** | ***nosZ***  **OTU**  **104** | ***nosZ***  **OTU**  **040** |
| --- | --- | --- | --- | --- | --- | --- | --- | --- | --- | --- | --- | --- | --- | --- | --- | --- |
| **Combined qPCR and OTU abundance (%)** | 0.62 | 0.40 | 0.31 | 0.23 | 0.22 | 0.19 | 0.14 | 1.03 | 0.48 | 0.46 | 0.32 | 0.32 | 0.23 | 1.11 | 0.27 | 0.24 |
| **Den** | - | - | - | -0.71 | - | - | - | - | - | - | - | - | - | - | -0.83 | - |
| **Dn** | - | - | -0.76 | - | - | - | - | - | - | 0.76 | - | - | -0.74 | - | **-**0.74 | 0.83 |
| **Dw** | - | - | - | - | - | - | - | - | - | - | - | - | - | - | - | - |
| **NO_3_^-^** | - | - | - | -0.83 | -0.71 | - | - | - | **-**0.79 | - | - | - | - | - | **-0.86** | - |
| **NH_4_^+^** | - | - | - | - | - | - | - | - | - | - | - | - | - | - | - | - |
| **PO_4_^3-^** | - | -0.76 | - | -0.78 | -0.79 | - | - | - | - | - | - | - | - | - | - | - |
| **°C** | - | - | - | - | - | - | - | - | - | - | - | - | - | - | - | - |
| **O_2_** | - | - | - | - | - | - | - | - | - | - | - | - | - | 0.81 | - | 0.74 |
| **Porosity** | - | - | **0.91** | - | - | - | - | - | - | -0.86 | - | - | - | - | - | **-0.91** |
| **LOI** | - | - | **0.91** | - | - | - | - | - | 0.81 | -0.81 | - | - | - | - | **-** | **-**0.83 |

**Supplementary Figure 1.** Phylogenetic classification to phylum level of 16S rRNA genes from lakes Lehee, Suolijärvi, Ormajärvi and Pääjärvi profundal samples.


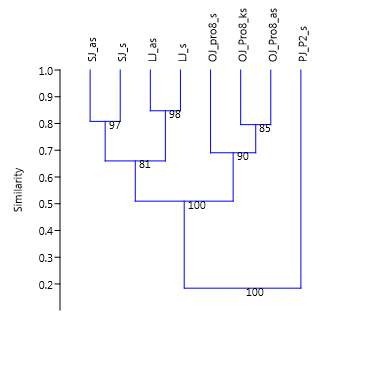


*nirS*


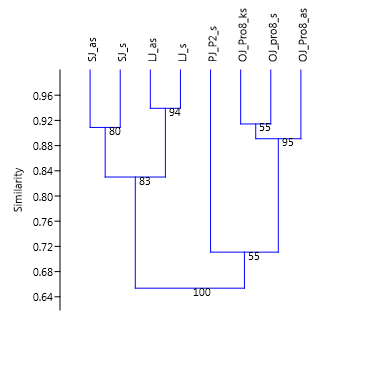


*nosZ*


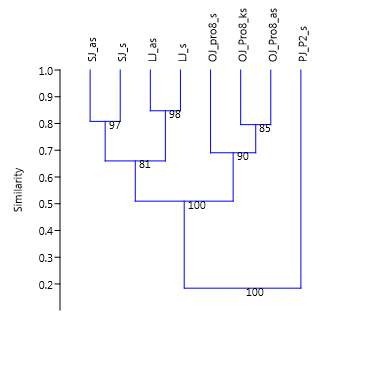


*nirK*

Pääjärvi

Ormajärvi

Suolijärvi

Lehee

**Lake**

Early-summer

Mid-summer

Autumn

**Season**

**Supplementary Figure 2.** UPGMA - clustering analysis of *nirS*, *nirK* and *nosZ* functional gene communities based on Bray Curtis distance matrix. The numbers at the nodes indicate percentages of occurrence in 1000 bootstrapped cluster dendrograms.

**Supplementary Figure 3.** Seasonal variations in *nirS, nirK and nosZ* gene relative abundances along the depth transect in Lake Ormajärvi.

**Supplementary Figure 4.** Seasonal variations in *nirS/nirK* ratios and denitrification rates (black diamonds) along the depth transect in Lake Ormajärvi.
